# Biochemical markers identify influences on bone and cartilage degradation in osteoarthritis - the effect of sex, Kellgren-Lawrence (KL) score, Body Mass Index (BMI), oral salmon calcitonin (sCT) treatment and diurnal variation

**DOI:** 10.1186/1471-2474-11-125

**Published:** 2010-06-17

**Authors:** MA Karsdal, I Byrjalsen, AC Bay-Jensen, K Henriksen, BJ Riis, C Christiansen

**Affiliations:** 1 Nordic Bioscience A/S, Herlev, DK-2730 Herlev, Denmark

## Abstract

**Background:**

Osteoarthritis (OA) involves changes in both bone and cartilage. These processes might be associated under some circumstances. This study investigated correlations between bone and cartilage degradation in patients with OA as a function of sex, Kellgren-Lawrence (KL) score, Body Mass Index (BMI), oral salmon calcitonin (sCT) treatment and diurnal variation.

**Methods:**

This study was a 2-week, double-blind, double-dummy, randomized study including 37 postmenopausal women and 36 men, aged 57-75 years, with painful knee OA, and a KL-score of I - III. Subjects were allocated to one of three treatment arms: 0.6 mg or 0.8 mg oral sCT, or placebo given twice-daily for 14 days. Correlations between gender, KL score, or BMI and the bone resorption marker, serum C-terminal telopeptide of collagen type I (CTX-I), or the cartilage degradation marker, urine C-terminal telopeptide of collagen type II (CTX-II) were investigated.

**Results:**

At baseline, biomarkers indicated women with OA experienced higher bone and cartilage degradation than men. CTX-I levels were significantly higher, and CTX-II levels only marginally higher, in women than in men (*p *= 0.04 and *p *= 0.06, respectively). Increasing KL score was not correlated with bone resorption, but was significantly associated with the cartilage degradation CTX-II marker in both men and women (*p *= 0.007). BMI was significantly and negatively correlated to the bone resorption marker CTX-I, r = -0.40 (*p *= 0.002), but showed only a borderline positive correlation to CTX-II, r = 0.25 (*p *= 0.12). Before morning treatments on days 1 and 14, no correlation was seen between CTX-I and CTX-II in either the sCT or placebo group. However, oral sCT and food intake induced a clear correlation between these bone and cartilage degradation markers. Four hours after the first sCT dose on treatment days 1 and 14, a significant correlation (r = 0.71, *p *< 0.001) between changes in both CTX-I and CTX-II was seen. In the placebo group a weakly significant correlation between changes in both markers was found on day 1 (r = 0.49, *p *= 0.02), but not on day 14.

**Conclusion:**

Bone resorption was higher in females than males, while cartilage degradation was correlated with increasing KL-score and only weakly associated with BMI. Bone and cartilage degradation were not correlated in untreated individuals, but dosing with oral sCT with or without food, and a mid-day meal, decreased bone and cartilage degradation and induced a correlation between both markers. Changes in bone and cartilage markers may aid in the identification of potential new treatment opportunities for OA.

**Trial Registration:**

Clinical trial registration number (EUDRACT2006-005532-24 & NCT00486369)

## Background

Bone and cartilage degradation are normally tightly coupled in the pathogenesis of osteoarthritis (OA), as subchondral bone turnover, sclerosis of the subchondral plate, trabecular thinning and articular cartilage loss are coexisting macroscopic changes [1]. Experimental and clinical observations suggest that the structural integrity of articular cartilage is dependent on normal subchondral bone turnover, intact chondrocyte function and ordinary biomechanical stresses [2,3]. An increasing line of evidence suggests that there are strong inter-relationships between the subchondral bone and the articular cartilage [1]. At present it is neither well-documented nor understood which parameters are initiators or drivers of the disease.

For the majority of patients, the etiology of OA is not known. Some evidence indicates that OA could be related to sex hormones, as decreased estrogen levels in both animal models and women are associated with increased cartilage degradation, in addition to the well-described increase in bone turnover [4-8]. Among the other known risk factors of OA are increasing age, sex, significant trauma, obesity and the resulting excessive loading, altered gait and altered biomechanics (e.g. varus or valgus deformity) [9-17]. This long list of risk factors for the initiation and progression of OA and physiological mechanisms affecting both bone and cartilage turnover are currently under investigation by several independent research groups using different approaches and technologies.

Biochemical markers of tissue turnover are increasingly used in both basic and clinical research, for diagnostic, prognostic and efficacy purposes [18,19]. In addition, such markers may provide additional information for understanding the pathology of disease. Osteoclast-mediated bone resorption involves the cysteine protease cathepsin K, which degrades the major protein in bone, type I collagen [20]. Cleavage of type I collagen results in release of C-terminal telopeptide of collagen type I (CTX-I) [20,21], which has been used extensively as a surrogate measure of bone resorption for *in vitro*, preclinical and clinical studies [20,22]. Cartilage degradation can be assessed using the C-terminal telopeptide of type II collagen (CTX-II), which is a Matrix Metalloproteinase (MMP)-generated fragment released during cartilage degradation in both inflammatory arthritis and OA [23,24]. CTX-II levels can predict loss of cartilage integrity, and are associated with OA and progression of OA [12,20,25].

An optimal approach to countering progression of OA may involve targeting the cells of both bone and cartilage. Calcitonin, a 32-amino-acid peptide, which possesses approved anti-resorptive abilities [26-32], has been shown to have positive effects on both osteoclasts and chondrocytes [33]. It has been shown to counter the progression of OA in preclinical models [34-38], and has shown promise in preliminary clinical settings [31,39-41]. Calcitonin administration was previously limited to subcutaneous or intra-nasal routes [30]. However a new oral formulation was recently presented [30,31] which has been proven safe and efficacious in a 3-month phase II study including postmenopausal women [30,31,42-45], and a dose of 0.8 mg taken bid is currently being assessed in phase III trials in OA.

The aim of the current investigation was to use CTX-I and CTX-II levels from OA patients taking part in a 2-week phase I study to assess correlations between bone and cartilage degradation in patients with OA as a function of sex, Kellgren-Lawrence (KL) score, Body Mass Index (BMI), oral sCT treatment and diurnal variation.

## Methods

### Drug substance

The study drug was an oral formulation of sCT consisting of the peptide hormone and 5-CNAC, {8-(N-2-hydroxy-5-chloro-benzoyl)-amino-caprylic acid)} a unimolecular enhancer of gastrointestinal peptide absorption. The moiety was designed to deliver sCT orally without significant enzymatic hydrolysis or alteration of the biological characteristics of sCT. When 5-CNAC is administered orally in combination with sCT, gastrointestinal absorption of sCT is markedly enhanced in rodents, monkeys and humans. The sCT-5-CNAC formulation was provided by Novartis, Basel, Switzerland.

The active tablets contained either 0.6 mg or 0.8 mg of sCT with 200 mg of 5-CNAC and were non-identical in appearance. Two different placebo tablets were used, each of which appeared identical to one of the sCT doses. Neither placebo tablet contained 5-CNAC or sCT. Due to the double-dummy design, participants took 2 tablets in the morning and 2 tablets in the evening before dinner. Tablets were taken with 50 ml of water.

### Study design

The data are a secondary analysis of, [46], a phase-I, 2-week, placebo-controlled, double-blind, double-dummy, randomized, gender-stratified study including 73 subjects,. Of these, 37 were postmenopausal women and the remaining 36 were men, previously reported (osteoarthritis and cartilage, in press). The study subjects fulfilled the inclusion criteria of being generally healthy, aged 57-75 years, and the women had passed a natural or surgical menopause at least 2 years before entering the study. At inclusion the subjects had painful OA of at least one knee, i.e. had knee pain for most days of the previous month, and experienced either morning stiffness of less than 30 minutes, or had crepitus. Additionally, the painful knee had to have a KL index score of I-III. The subjects did not have diseases or receive medications, such as bisphosphonates, known to affect bone metabolism.

After consenting, the subjects were randomized to one of 3 treatment arms: treatment with 0.6 mg of sCT (12 women and 12 men), or 0.8 mg of sCT (13 women and 13 men), or placebo (12 women and 11 men). Each treatment was given twice-daily for 14 days. The morning dose was administered between 07:00 and 08:00 at least 30 minutes before breakfast, except on days 1 and 14 when the 08.00 dose was followed by fasting for 5 hours. The second dose was administered 30 minutes (days 2 - 13 inclusive) or 60 minutes (days 1 and 14) before evening dinner.

The study period included an initial screening visit and a treatment period of 14 days. Pharmacokinetic/pharmacodynamic (PK/PD) assessments were made at baseline, and prior to treatments on treatment days 1 and 14. Visits were scheduled on day 1, on day 2, after 1 week of treatment, on treatment day 14 for a PK/PD assessment, and on day 15. On the first day of treatment, the subjects arrived at the clinic after an overnight fasting period and received the first dose of study drug at 08:00. The subjects remained fasting until lunch was served at 13.00. A light meal was eaten at 15:30. The second dose was given at 17:00, and dinner was served 1 hour later at 18:00. The same dosing and meal schedule was applied on treatment day 14.

For PD evaluation on treatment days 1 and 14 blood samples were collected immediately prior to dosing (time 0 hrs), and at intervals of 1/2, 1, 2, 4, 6 and 8 hrs after morning dose and, for the pre-dinner dose, immediately prior to dosing (time 0 hrs), and at the intervals of 1/2, 1, 2, and 4 hrs post-dose. Urine samples were collected pre-dose and at 2, 4, 6, 8, 13, and 15 hours after the morning dose on treatment days 1 and 14. All samples were stored at -20°C until analysis.

The study was conducted in accordance with Helsinki Declaration II, version II. The study was approved by the local Danish Ethical Committees, and conducted in Ballerup and Vejle, Denmark. The study was registered as EUDRACT: 2006-005532-24

### Biochemical assays

Serum CTX-I was determined by the Serum CrossLaps One Step ELISA (IDS Nordic, Herlev, Denmark) [47]. Urine CTX-II was determined using the Urine Cartilaps ELISA (IDS Nordic, Herlev, Denmark). Urinary creatinine was measured by a routine chemistry method and used for calculation of creatinine-corrected urinary CTX-II concentrations.

### Statistical analysis

For correlations the 0.6 and 0.8 mg sCT groups were combined into one sCT group. To assess changes in serum CTX-I and urinary CTX-II after dosing, values were calculated for each person and expressed relative to the pre-dose value. Data of serum CTX-I, urinary CTX-II, and relative levels of urinary CTX-II were logarithmically transformed to obtain normality and symmetry of variances.

Analysis of variance (ANOVA) was used for comparison of the continuous variables of baseline characteristics between the three treatment groups. Fisher's exact test was applied for assessment of the proportions of subjects classified in KL-score groups between the three treatment groups. The general linear ANOVA model was applied for assessment of differences of baseline levels of serum CTX-I and urinary CTX-II between genders, and for assessment of their relationship with KL-scores and BMI. In these models the baseline value of the biochemical marker was used as a response variable and KL-score and gender or BMI and gender were included as fixed effects. Spearman rank correlation coefficient was used to assess the correlation between the levels of serum CTX-I and urinary CTX-II in placebo and the sCT-treated subjects before and during treatment.

A difference was considered significant if the *p*-value was less than 5%. All statistical calculations were performed using the SAS software package (release 9.1, SAS Institute Inc., Cary, NC, USA).

## Results

The demographic characteristics are presented in Table 1. No relevant differences within the individual gender treatment groups were present, and all three treatment groups had similar numbers of patients with KL I, II and III scores.

**Table 1:** Baseline demographic characteristics of study population

	Placebo	0.6 mg sCT	0.8 mg sCT	*p*-value
Females	*n *= 12	*n *= 12	*n *= 13	-
Males	*n *= 11	*n *= 12	*n *= 13	

Age (yrs)	65.7 (4.1)	64.5 (4.4)	66.7 (4.1)	0.43
	64.6 (3.7)	65.3 (1.4)	64.0 (4.0)	0.58

YSM (yrs)	21 (8)	15 (6)	17 (6)	0.14
	-	-	-	-

Height (cm)	168.2 (5.1)	167.2 (7.7)	161.9 (5.5)	0.03
	177.8 (7.1)	176.9 (6.1)	175.5 (7.9)	0.73

Weight (kg)	76.4 (15.2)	73.4 (10.3)	78.7 (13.5)	0.61
	89.0 (15.2)	85.2 (9.0)	91.9 (17.0)	0.51

BMI (kg/m^2^)	27.0 (5.6)	26.4 (4.1)	29.9 (3.9)	0.13
	28.2 (5.1)	27.2 (2.4)	29.7 (4.4)	0.32

KL Index	I: 7 (30%)	I: 7 (29%)	I: 9 (35%)	0.80
	II: 8 (35%)	II: 11 (46%)	II: 12 (46%)	
	III: 8 (35%)	III: 6 (25%)	III: 5 (19%)	

Values given are mean (SD). Years since menopause (YSM), body mass index (BMI), Kellgren-Lawrence (KL). The *p*-value column refers to the significance of the comparison of the three treatment groups.

**Figure 1:**
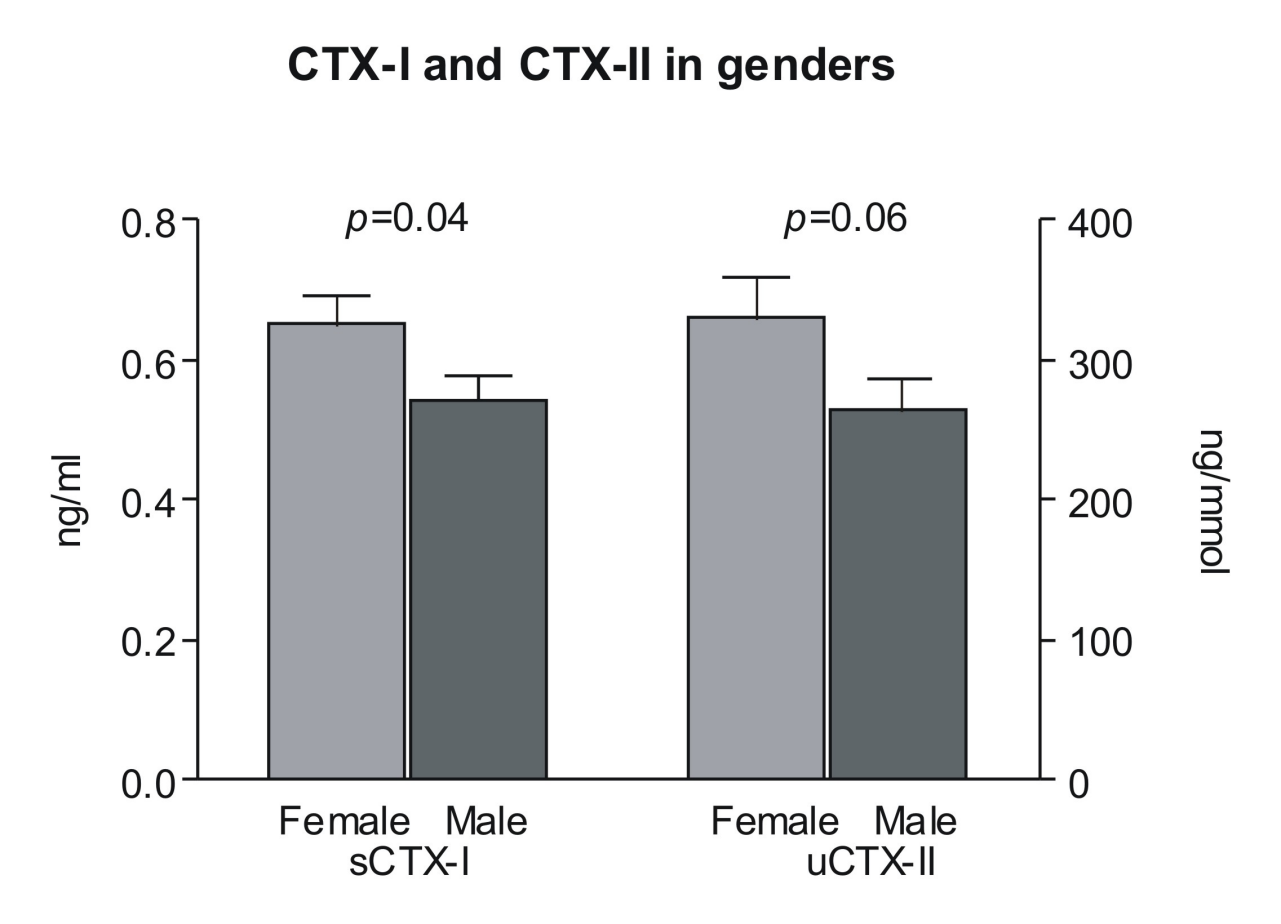
**Baseline concentrations of serum CTX-I and urinary CTX-II measured in samples collected immediately prior to first dosing at treatment day 1 stratified according to gender**. Values shown are geometric mean +1 SEM.

Baseline measurements of CTX-I and CTX-II showed no significant differences between the treatment groups (Table 2). However, women had higher serum CTX-I baseline levels than men, indicating higher bone resorption: this difference was statistically significant (*p *= 0.04) (Figure 1). While the baseline urine CTX-II levels in women were slightly higher than in men, the difference was of borderline significance (*p *= 0.06).

**Table 2:** Baseline values of serum CTX-I and urinary CTX-II

	Placebo	0.6 mg sCT	0.8 mg sCT	*p*-value
Females				

sCTX-I (ng/ml)	0.758 (0.536-1.073)	0.669 (0.503-0.891)	0.555 (0.378-0.817)	0.09

uCTX-II (ng/mmol)	303 (161-570)	410 (268-626)	293 (192-449)	0.21

Males				

sCTX-I (ng/ml)	0.539 (0.413-0.704)	0.588 (0.353-0.981)	0.504 (0.340-0.747)	0.64

uCTX-II (ng/mmol)	278 (200-387)	256 (159-411)	263 (153-455)	0.91

Values given are geometric mean (± 1SD range). The *p*-value column refers to the significance of the comparison of the three treatment groups.

**Figure 2:**
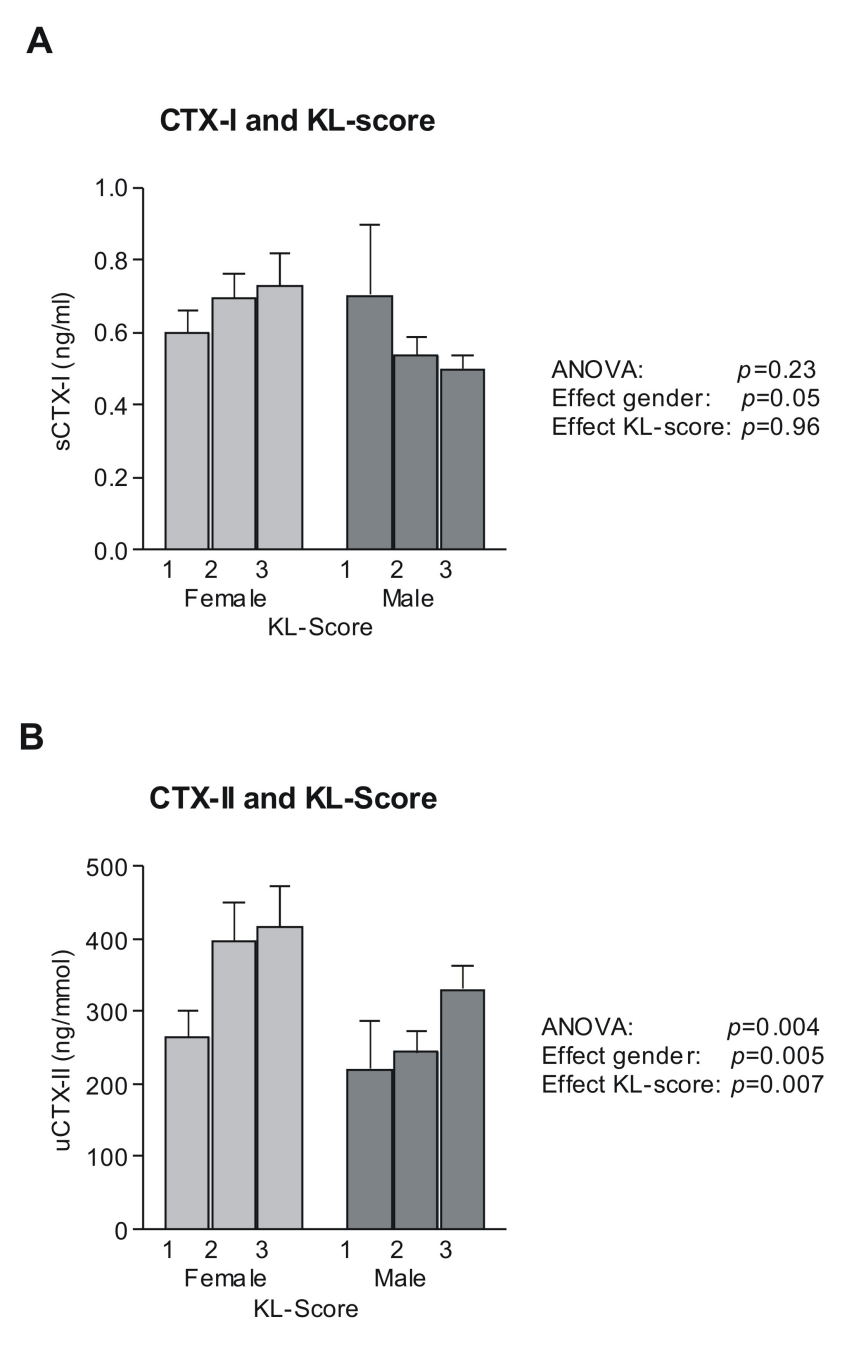
**Baseline concentrations of A) serum CTX-I and B) urinary CTX-II, corrected to creatinine, measured in samples collected immediately prior to first dosing at treatment day 1 and stratified according to KL-score index for each gender**. Values shown are geometric mean +1 SEM.

At baseline, correlations between CTX-I and KL-score were not found (Figure 2A). Although a trend towards an increase in CTX-I with increasing OA severity was seen in women, it was completely absent in men. On the other hand, CTX-II levels were significantly correlated to increasing KL-score in both men and women (*p *= 0.007) (Figure 2B). The effect was more pronounced in women than in men.

**Figure 3:**
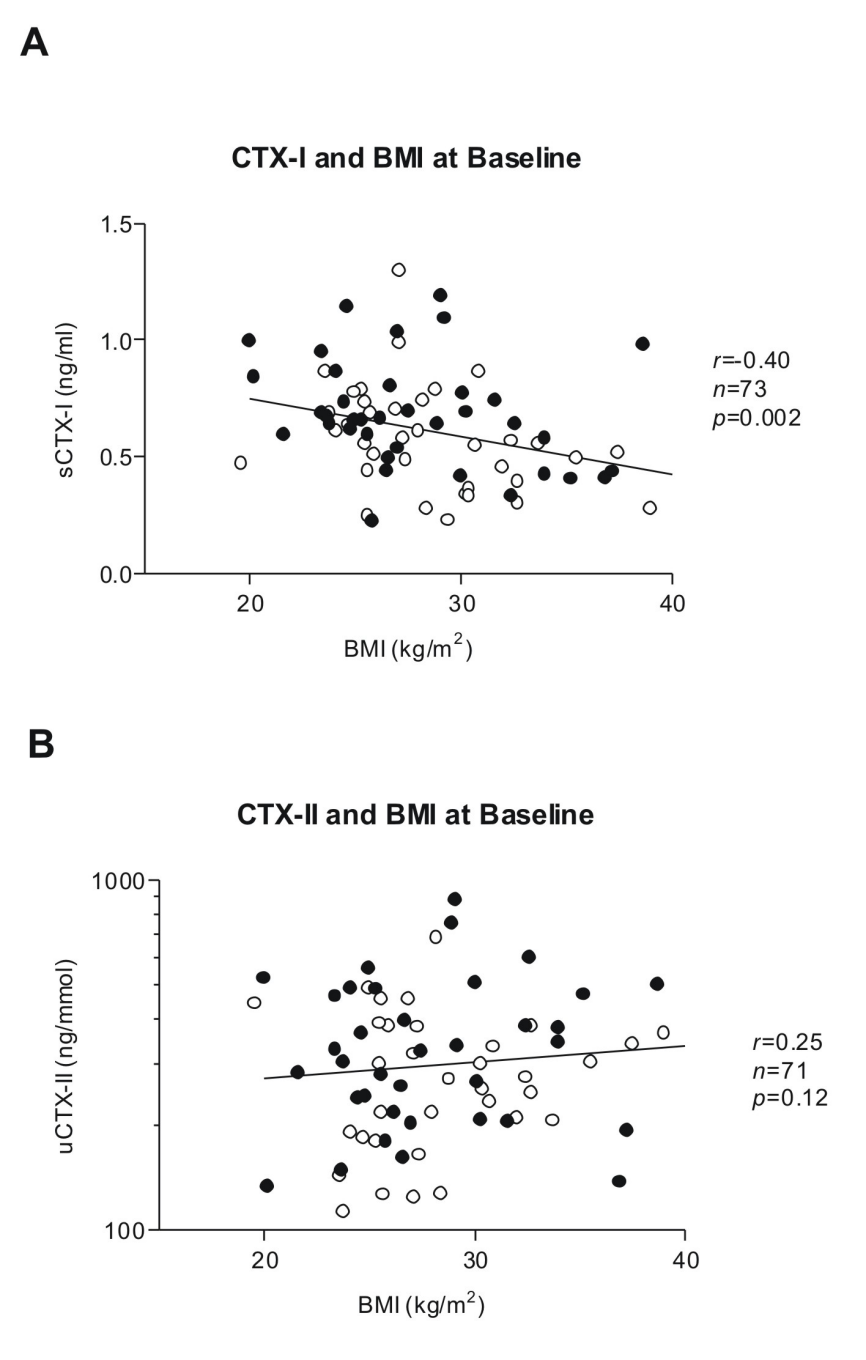
**Relationship between baseline concentrations of BMI and A) serum CTX-I and B) urinary CTX-II measured in samples collected immediately prior to first dosing at treatment day 1**. Females: filled circles; Males: open circles.

Investigations of the correlation between BMI and the two markers revealed that CTX-I was negatively correlated to BMI, r = -0.40 (*p *= 0.002) (Figure 3A), whereas CTX-II showed a weak and non-significant positive correlation with BMI, r = 0.25 (*p *= 0.12) (Figure 3B). No differences in correlations between BMI and the markers were observed between genders.

**Figure 4:**
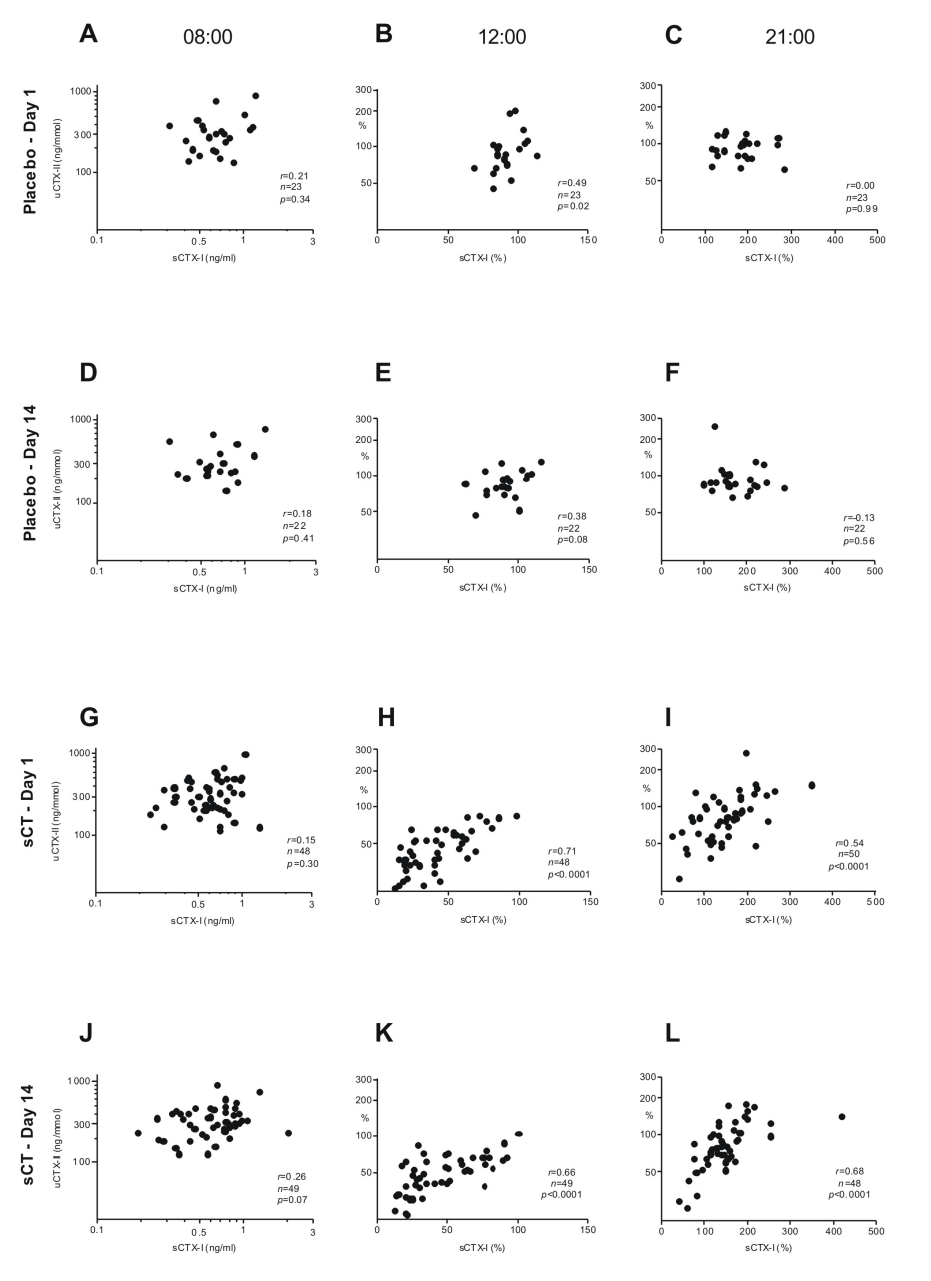
**Relationship between serum CTX-I and urinary CTX-II (corrected to creatinine) in untreated subjects and subjects treated with sCT**. A) & C) Concentrations in samples collected at 08:00 immediately prior to first dosing at treatment day 1. B) & D) Concentrations measured in samples collected at 12:00, i.e. 4 hours post-morning dose, relative to pre-dose at 08:00. C) & I) Concentrations measured in samples collected at 21:00, i.e. 4 hours after the pre-dinner dose, relative to pre-dose at 17:00. D) & J) Concentrations in samples collected at 08:00 immediately prior to dosing at treatment day 14. E) & K) Concentrations measured in samples collected at 12:00, i.e. 4 hours post morning dose, relative to pre-dose at 08:00. F) & L) Concentrations measured in samples collected at 21:00, i.e. 4 hours post pre-dinner dose, relative to pre-dose at 17:00.

The correlation between changes in CTX-I and CTX-II in response to treatment was investigated on both treatment days 1 and 14 of the study. Before morning dosing on treatment day 1, no correlation was seen between the two markers in either the placebo or the combined 0.6 and 0.8 mg sCT dose groups placebo or sCT groups. (Figure 4A &4G). These findings were similar at treatment day 14 (Figure 4D &4J), although a trend towards a correlation was seen in the sCT group (r = 0.26, *p *= 0.07). At noon, i.e. 4 hours after dosing, a significant correlation (r = 0.71, *p *< 0.001) between changes in CTX-I and CTX-II was seen in the sCT group on both days 1 and 14, indicating reduced degradation of both bone and cartilage, whereas in the placebo group a weakly significant correlation was found on day 1 (r = 0.49, *p *= 0.02), but not on day 14 (Figure 4B, E, H, &4K). Finally, the evening measurements (4 hours after the second dosing, 3 hours after dinner), showed a clear and significant (r = 0.54, *p *< 0.0001) correlation between changes in CTX-I and CTX-II in the sCT group on both days, again indicating suppression of bone and cartilage degradation. No correlation was observed in the corresponding placebo groups regardless of the day (Figure 4C, F, I, &4L).

**Figure 5:**
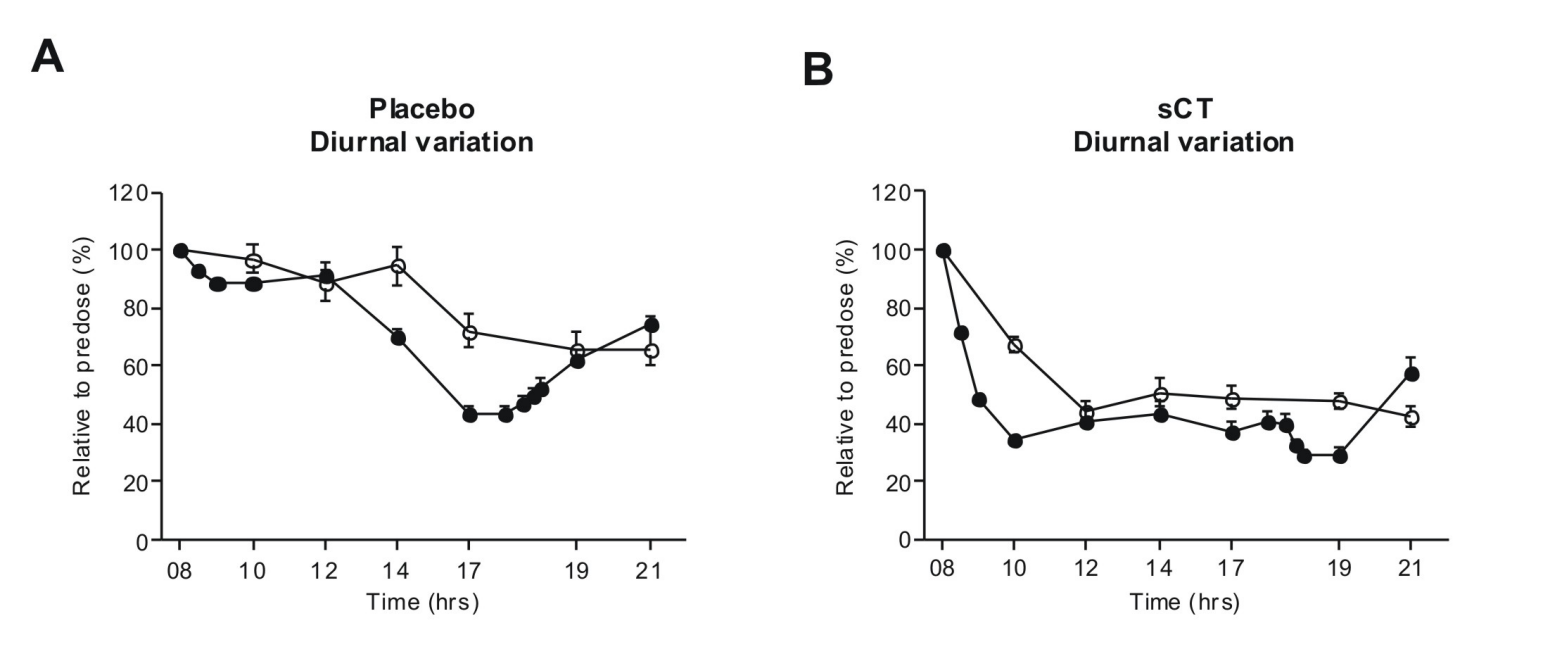
**Time course during the day of concentrations serum CTX-I (filled circles) and urinary CTX-II (open circles) in A) untreated subjects and B) subjects treated with sCT**. Values shown are concentrations relative to pre-dose at 08:00 and given as mean ± 1 SEM for serum CTX-I and geometric mean ± SEM for urinary CTX-II. A meal was given at 13.00 hours and a light meal at 15.30 as described in the material and methods. Dinner was served at 18.00. Modified from [46].

CTX-I displayed marked diurnal variation, in which a more than 50% decrease in levels was seen as a response to intake of lunch in the placebo group. CTX-II displayed lower levels of diurnal variation in the placebo group; however the kinetics of the changes were comparable to those in CTX-I (Figure 5A), indicating that CTX-II levels also are affected by the lunchtime meal. In the combined sCT treatment group analysis, robust reductions in both CTX-I and CTX-II were observed shortly after the morning dose, albeit with a more rapid reduction in CTX-I than CTX-II (Figure 5B). The marker levels remained suppressed throughout the day and early evening. The data are modified from [46].

## Discussion

Ample evidence of a concomitant breakdown of bone and cartilage is found in the pathogenesis of OA in both clinical [11,48] and preclinical settings [49-51]. This present study elaborates on this potential relationship by demonstrating that: 1) Women have higher bone resorption and cartilage degradation than men, 2) The KL score is significantly associated with cartilage, but not bone, degradation markers, 3) BMI is significantly and negatively correlated to bone resorption, but only shows a borderline positive correlation to cartilage degradation, 4) Untreated individuals do not display an obvious correlation between bone and cartilage degradation markers, whereas calcitonin treatment and food intake induced a clear correlation between these markers.

The present investigation indicated that women have higher cartilage degradation compared with men in all three KL categories. Such OA may be referred to as a systemic, metabolic form of OA [1], which is supported by our finding that bone resorption, indicated by CTX-I levels, was higher in women than in men. Similar findings have been shown for postmenopausal women in whom OA is more pronounced, than in the pre-menopausal population [4,52]. Furthermore, these findings also correlate with the notion that prevalence of OA is higher in women, although there are subpopulations or individual joints where the opposite appears to be true [10,53-55]. An alternative approach to investigating the correlation between bone and cartilage turnover and deterioration in the pathogenesis in OA is through analysis of the response to various treatments in both preclinical and clinical studies. In particular reference to the notion that cartilage degradation was higher in females compared to that of males, we have previously demonstrated the therapeutic benefit of the selective estrogen-receptor modulator (SERM), levormeloxifene, in a phase II trial assessing the prevention of both bone loss and cartilage degradation during a 12 month period [8]. Levels of both CTX-II and CTX-I, as markers of cartilage and bone degradation respectively, decreased by approximately 50% in the treatment group compared with baseline, and CTX-II levels were restored to premenopausal levels. Similarly, another group found that cartilage degradation in 384 postmenopausal women was significantly lower in women receiving hormone replacement therapy (HRT) compared with women not receiving HRT [4]. These data are in alignment with the recent analysis of the Women's Health Initiative studies, which documented that women taking estrogen had less total joint surgery compared with women taking placebo [56]. The findings, taken together with the previously mentioned over-representation of OA in women compared with men, support the hypothesis that sex hormones are related to the incidence of OA. In addition, experimental studies in monkeys [57,58] and rats [59] have shown that estrogen depletion results in increased bone turnover and accelerated cartilage breakdown. These combined findings enable a deeper understanding of the rationale of the elevated CTX-II level in females compared to that of males.

In the current study a clear correlation between markers of bone and cartilage degradation was seen after treatment with calcitonin, but not in untreated individuals. This is interesting, as suggest that only in some situation a correlation between bone and cartilage turnover is observed. To highlight a causal relationship between bone and cartilage turnover in the pathogenesis of osteoarthritis, Duong and colleagues investigated the effects of intravenous bisphosphonate treatment on bone turnover and development of OA in a traumatic model of OA, the anterior cruciate ligament model (ACLT)[3,50]. Bisphosphonate treatment resulted in a 50% decrease in disease severity scores, demonstrating the importance and coupling between bone and cartilage health. Additional support for the role of the subchondral bone in OA was found in studies examining the effect of calcitonin in ACLT dogs [36,38]. Calcitonin significantly affected trabecular structure and prevented subchondral bone resorption and trabecular thinning, which was speculated to be a major factor in the reduced cartilage degradation [32,35,38]. However, the mode of action of calcitonin may be different from other anti-resorptives, as calcitonin was demonstrated to have both direct and indirect action on articular cartilage [30-32,35,36,38,40,60-64], which may complicate the interpretation of this findings. In addition, even in the face of the diversity of animal models that exists and have been employed in both pharmacological research and in the understanding of the pathogenesis of OA, cautions must be applied. Animal models may not adequately reflect i) the diversity of the disease status ii) initiation or iii) progression in man, which currently is heavily debated in the field [65,66].

Bone and cartilage metabolism has been shown to be correlated in many studies of anti-resorptive treatments [1,31]. However, recently tibolone, a synthetic steroid with estrogenic, androgenic, and progestogenic properties, was shown to strongly inhibit bone resorption by 60% with no positive effects on cartilage degradation [67,68]. This further highlights that bone and cartilage turnover may be linked under some pathological and physiological processes and conditions, but not others.

Taken together, this increasing line of evidence points toward some anti-resorptive treatments, such as bisphosphonates, calcitonin, estrogen or SERMs, having positive effects on both cartilage and bone degradation, possibly due to a tight coupling between these compartments. Whatever the underlying reason is for the concomitant degradation, a dual-action OA therapy that can protect both bone and cartilage from deteriorating further, would seem ideal. An interesting question is whether some of the anti-resorptives (ie, calcitonin and estrogen) may, in addition to their direct effects on osteoclasts and bone turnover, provide additional benefits by targeting chondrocytes directly.

Weight is considered an important risk factor for OA [69,70]. In the present study we found a negative correlation between BMI and the marker of bone resorption, which could in part be due to the peripheral estrogen production of adipose tissue [71-73]. This may explain the only borderline positive correlation between cartilage degradation and BMI, which was slightly surprising since OA and obesity are correlated in many cases [69,70]. Not surprisingly, cartilage degradation was strongly associated with KL-score, in contrast to bone resorption [25,74].

Diurnal variation is a well-established and important parameter of bone turnover, with bone resorption marker levels decreasing by approximately 50% after food intake compared with those of fasting individuals, and an equally large increase in bone resorption marker levels occurring during the night [75-77]. In the present study we found a similar decrease in bone resorption after meal intake at 13.00 hours, approximately 60%, (CTX-I). A lower decrease in cartilage degradation after meal intake of approximately 30% was observed. It has previously been suggested that hormones impacted by food intake may positively affect bone metabolism. An example of a hormone affected by food intake is the glucagon-like peptide-2 (GLP-2), which has a positive effect on calcium metabolism and has been shown to be a strong physiological inhibitor of bone resorption [78]. GLP-2 was identified in a systematic screening, using CTX-1, of hormones related to postprandial regulation of bone resorption [78]. In alignment, other hormones that affect cartilage metabolism may be identified by a systematic screening of the response of bone resorption and cartilage degradation biochemical markers.

This novel oral form of sCT have been investigated in other Phase I and II clinical settings, [30,31,46,79-83]. Whether this novel oral form of calcitonin may provide clinical benefit to patients remain to be presented in pivotal clinical trials. These Phase III clinical trials are currently ongoing.

## Conclusion

Biochemical makers in this study show that bone resorption is correlated with female gender, while cartilage degradation is correlated with increasing KL-score and only weakly correlated with BMI. Furthermore, in untreated patients with OA, bone and cartilage degradation is not correlated. However, a lunchtime meal in untreated patients, and treatment with oral sCT, regardless of whether food is taken or not, prevent degradation of both tissues to varying degrees, and produce a correlation between CTX-I and CTX-II. Together with findings from other published studies, it seems that bone resorption and cartilage degradation are influenced to differing degrees by various physiological and pathological situations, as well as by food intake and hormone therapy. The use of biomarkers to assess and monitor degradation of these key tissues may aid in the early identification of treatment opportunities for OA.

## Abbreviations

OA: Osteoarthritis; sCT: Salmon calcitonin; GLP-2: glucagon-like peptide-2; CTX-I: C-terminal telopeptide of collagen type I; CTX-II: C-terminal telopeptide of collagen type I; PK: Pharmacokinetic; PD: Pharmacodynamic; AUC: Area under the curve; KL: Kellgren-Lawrence; BMI: Body mass index; MMP: Matrix Metallo Protease.

## Competing interests

Claus Christiansen, Bente J Riis and Morten A. Karsdal own stock in Nordic Bioscience. All other authors are full-time employees of Nordic Bioscience. Nordic Bioscience is involved in the development of oral sCT for the treatment of osteoporosis and osteoarthritis.

## Authors' contributions

MK had the original idea for the manuscript and drafted the first version. CC and BJR designed the study and participated in drafting the first version of the manuscript. IB performed statistical analysis and participated in reviewing the manuscript. KH and ACBJ participated in discussing and providing a final version of the manuscript. All authors read and approved the last version of the manuscript.

## Pre-publication history

The pre-publication history for this paper can be accessed here:

http://www.biomedcentral.com/1471-2474/11/125/prepub
